# Surgical management of large ventral hernias following damage control surgery: a retrospective comparison of laparoscopic IPOM plus and open repair techniques

**DOI:** 10.3389/jaws.2026.16917

**Published:** 2026-07-16

**Authors:** Marharyta Smirnova, Oleh Herasymenko, Mykhailo Koshikov, Yaroslav Haida, Alim Ulukhanov

**Affiliations:** Military Medical Clinical Centre of the Southern Region, Odesa, Ukraine

**Keywords:** abdominal wall reconstruction, combat surgical trauma, damage control surgery, pain, ventral hernia

## Abstract

Damage control surgery (DCS) has become the standard of care in the management of severe abdominal trauma, particularly in military settings. Although this approach improves survival, it frequently results in significant disruption of the anterior abdominal wall and subsequent development of large ventral hernias. Reconstruction in this context is technically challenging due to adhesions, distorted anatomy, and loss of normal tissue planes. While open retromuscular repair is generally preferred, it is often not feasible following DCS. Laparoscopic intraperitoneal on-lay mesh repair with primary fascial closure (IPOM plus) may offer an alternative. However, evidence in post-traumatic populations remains limited. This study aimed to compare the feasibility, safety, and short-term outcomes of laparoscopic IPOM plus and open repair techniques in patients undergoing ventral hernia repair after DCS. A retrospective cohort study was conducted at a single military centre between June 2022 and December 2025. A total of 79 male patients with large ventral hernias following abdominal shrapnel injuries were included. Patients underwent either open repair (sub-lay or on-lay, n = 46) or laparoscopic IPOM plus repair (n = 33). Baseline characteristics were comparable between groups. Early postoperative outcomes within 90 days were analysed, including operative time, length of hospital stay, postoperative pain, time to recovery of bowel function, and complication rates. Laparoscopic IPOM plus repair was feasible in all cases, with successful primary fascial closure achieved in every patient. Operative time did not differ significantly between groups. The laparoscopic approach was associated with a significantly shorter hospital stay (p < 0.001), lower early postoperative pain on postoperative days 1 and 2 (p < 0.05), and earlier recovery of bowel function (p < 0.01). Rates of postoperative complications, including seroma formation, were lower following laparoscopic repair but did not reach statistical significance. No surgical site infections, reoperations, or readmissions were observed in either group. In patients with large ventral hernias following DCS, laparoscopic IPOM plus repair appears to be a feasible and safe alternative to open reconstruction. It is associated with improved early postoperative recovery without an increase in short-term complications. Further prospective studies with long-term follow-up are required to confirm these findings.

## Introduction

**FIGURE 1:**
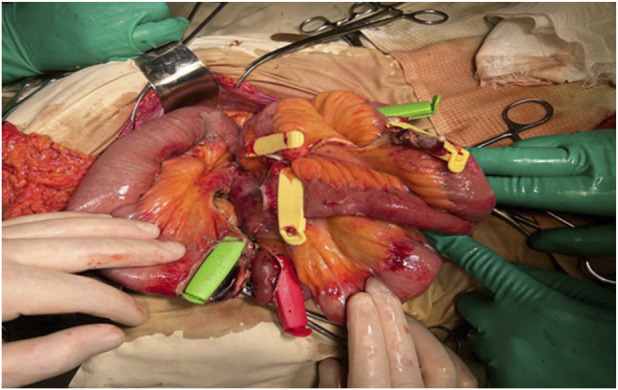
Application of umbilical clips to segments of the small intestine during the first stage of damage control surgery. Photo taken by the authors. Licensed under CC-BY 4.0.

**FIGURE 2:**
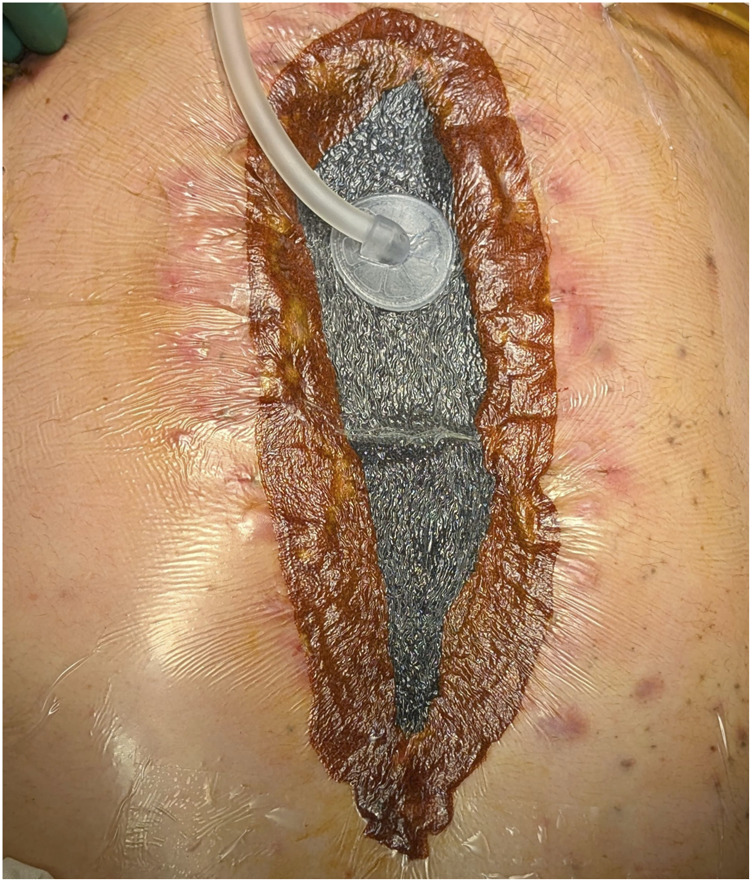
Temporary abdominal closure with application of an abdominal VAC system during evacuation between the first and second stages of damage control surgery. Photo taken by the authors. Licensed under CC-BY 4.0.

**FIGURE 3:**
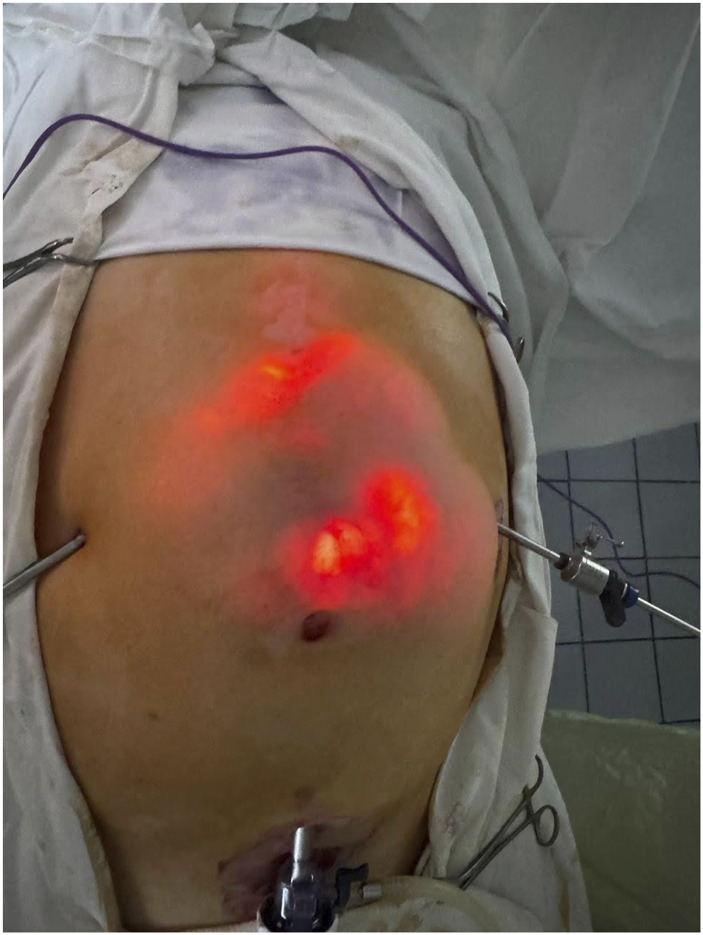
External view of a ventral hernia during laparoscopy with established pneumoperitoneum. Photo taken by the authors. Licensed under CC-BY 4.0.

Since the beginning of the full-scale war in Ukraine, a substantial number of patients have sustained abdominal gunshot and shrapnel injuries. Damage control surgery (DCS) (Figure 1) has become a standard life-saving approach for patients with severe abdominal trauma [1, 2], particularly in the setting of military conflict and high-energy injuries. While DCS significantly improves survival, repeated laparotomies, temporary abdominal closure (Figure 2), and secondary wound healing frequently result in severe disruption of the anterior abdominal wall. Consequently, many survivors develop large and complex ventral hernias that require delayed elective reconstruction (Figure 3).

In this study, hernia defect size was classified according to the European Hernia Society (EHS) classification for incisional abdominal wall hernias, which defines defect width as W1 (<4 cm), W2 (≥4–10 cm), and W3 (≥10 cm) [3]. All patients included in the present cohort had W3 defects, corresponding to large ventral hernias.

Surgical repair of ventral hernias following DCS is technically demanding. Extensive adhesions, loss of normal tissue planes, distortion or absence of the posterior rectus sheath, and trauma-related anatomical alterations often limit the feasibility of conventional reconstructive techniques. Although retromuscular (sub-lay) repair is generally regarded as the preferred approach for large ventral hernias [4, 5], it is frequently not achievable in patients after multiple emergency laparotomies. In such situations, on-lay repair is commonly employed; however, in this complex population, open techniques may be associated with increased surgical trauma and prolonged postoperative recovery.

Laparoscopic Intraperitoneal On-lay Mesh with primary fascial defect closure (IPOM plus) repair represents an alternative strategy that may offer reduced access trauma and faster functional recovery in selected patients [6], even in the presence of complex post-traumatic anatomy. Nevertheless, evidence guiding the optimal choice of surgical technique for ventral hernia repair after DCS remains limited. Most available studies focus on elective hernia repair in non-trauma populations [7, 8] and may not be directly applicable to patients following damage control surgery.

The aim of this study was to retrospectively compare the feasibility, safety, and short-term outcomes of laparoscopic IPOM plus and open repair techniques (on-lay and sub-lay) in patients undergoing elective ventral hernia repair after abdominal trauma managed with DCS.

## Materials and methods

### Study design and setting

This retrospective cohort study was conducted at the Military Medical Clinical Centre of the Southern Region in Odesa, Ukraine, between June 2022 and December 2025. The study included 79 male patients who sustained combat-related abdominal injuries and subsequently underwent elective surgical repair of ventral hernias. The minimum interval between the initial injury and the planned hernia repair was 6 months.

All patients sustained small bowel injuries, and some also had associated liver injuries without involvement of major blood vessels or bile ducts. Patients with more severe injury patterns, including major vascular or biliary tract injuries, were not included in the study.

Social habits, including smoking and alcohol consumption, were recorded at admission. No active alcohol use was reported at the time of surgery. Smoking was common in the cohort, and its prevalence was comparable between the study groups. Patients with significant comorbidities, such as diabetes mellitus, chronic cardiovascular insufficiency, chronic pulmonary disease, chronic kidney or liver disease, as well as chronic pain syndromes, were excluded in order to minimise potential confounding factors.

Demographic and clinical data, including age, body mass index (BMI), comorbidities, operative characteristics, and postoperative outcomes, were collected for all patients. Only patients with complete clinical and perioperative datasets were included in the final analysis.

### Patient groups

Patients were divided into two groups according to the surgical approach. From September 2023 to August 2025, ventral hernia repair in this patient population was performed exclusively using open techniques. In September 2025, laparoscopic IPOM plus repair was introduced into clinical practice. The surgical team had prior experience with this technique in elective ventral hernia repair in non-trauma patients. From October 2025 to December 2025, laparoscopic repair became the standard approach and was performed exclusively in this cohort. All procedures were carried out at the same institution under standardized perioperative management protocols.

A total of 46 patients underwent open surgery, while 33 patients were treated using a laparoscopic approach. The two groups were comparable with respect to baseline clinical characteristics including age, BMI, comorbidities, and injury profile (Table 1).

**TABLE 1:** Baseline characteristics of the study population.

Parameter	Open group (n = 46)	Laparoscopic group (n = 33)
Age, years (mean ± SD, range)	37.9 ± 9.2 (26–55)	35.7 ± 10.3 (20–55)
BMI, kg/m^2^ (mean ± SD, range)	22 ± 2.91 (18–26)	21.48 ± 3.21 (17–27)
Smoking, n (%)	21 (45.7%)	17 (51.7%)
Hernia defect size, cm (mean ± SD, range)	12.55 ± 2.38 (10.5–17)	13.03 ± 1.96 (10.5–16.5)
Associated liver injury	13 (28.3%)	9 (27.3%)

### Surgical techniques

In the open surgery group, retromuscular (sub-lay) repair was considered the preferred technique and was planned whenever feasible. Sub-lay repair was performed in 25 patients. However, in a substantial proportion of patients following damage control surgery, the retromuscular plane was partially or completely disrupted due to multiple laparotomies, extensive scarring, dense adhesions, or loss of the posterior rectus sheath. In such cases, safe dissection of the retromuscular space was not possible. When reconstruction of the retromuscular plane was not feasible, an on-lay mesh repair was performed. Overall, 21 patients in the open group underwent on-lay repair. The choice between sub-lay and on-lay repair was dictated by intraoperative anatomical findings rather than by surgeon preference.

**FIGURE 4:**
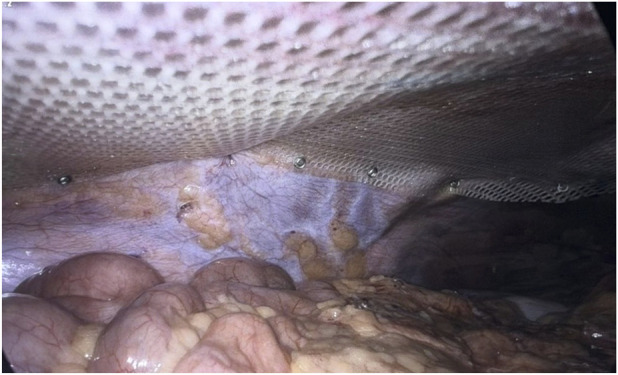
Intraperitoneal placement and circumferential fixation of a composite mesh using the IPOM plus technique. Photo taken by the authors. Licensed under CC-BY 4.0.

In the laparoscopic group, ventral hernia repair was performed using the IPOM plus technique. Primary fascial defect closure with non-absorbable sutures was achieved in all patients prior to mesh placement, ensuring restoration of the anterior abdominal wall continuity without undue tension. Subsequently, a composite mesh was positioned intraperitoneally with sufficient overlap and secured using circumferential fixation in accordance with established IPOM plus technique standards (Figure 4).

### Outcome measures

Given the limited and unequal follow-up duration between the study groups, long-term outcomes such as hernia recurrence and chronic pain were not evaluated. The analysis therefore focused on early postoperative outcomes within the first 90 days after surgery.

Postoperative outcomes included length of hospital stay, operative time, and the incidence of postoperative complications such as seroma, haematoma, surgical site infection, and postoperative intestinal obstruction. Postoperative pain intensity was assessed using the visual analogue scale (VAS). Recovery of bowel function was evaluated as the time to first postoperative bowel movement. The need for reoperation and hospital readmission within 90 days after surgery were also recorded.

Under current treatment conditions in Ukraine, length of hospital stay reflects not only medical recovery but also institutional requirements, as discharge was permitted only after complete wound healing and suture removal, full recovery of physiological functions, and confirmation that no further medical assistance was required.

All outcome data were collected retrospectively from institutional medical records.

### Statistical analysis

Statistical analysis was performed using IBM SPSS Statistics, version 27.0 (IBM Corp., Armonk, NY, USA). The distribution of continuous variables was assessed using the Shapiro–Wilk test. Continuous data are presented as mean ± standard deviation and were compared between groups using the independent samples t-test. Categorical variables are presented as counts and percentages and were analyzed using the χ^2^ test or Fisher’s exact test, depending on the expected cell frequencies. All statistical tests were two-sided. A p-value <0.05 was considered statistically significant. No missing data were identified in the study cohort.

## Results

All patients included in the analysis were male and had sustained combat-related abdominal shrapnel injuries managed with damage control surgery. The interval between the initial injury and elective ventral hernia repair was at least 6 months in all patients. Open ventral hernia repair was performed in 46 patients, while 33 patients underwent laparoscopic IPOM plus repair.

The open and laparoscopic groups were similar with respect to baseline demographic and clinical characteristics. Age, body mass index, smoking status, injury profile, hernia defect size, and the severity of intra-abdominal adhesions assessed by the Peritoneal Adhesion Index did not differ significantly between the groups (Table 1). All patients had W3 ventral hernia defects according to the European Hernia Society classification. No patients reported active alcohol consumption at the time of surgery, and no clinically relevant comorbidities were identified. In addition, no significant differences were observed regarding associated liver injury or the number of previous laparotomies.

### Operative characteristics

Operative characteristics are summarised in Table 2.

**TABLE 2:** Surgical characteristics and intraoperative details.

Parameter	Open group (n = 46)	Laparoscopic group (n = 33)
Time from injury to repair, month (mean ± SD, range)	8.72 ± 2.51 (6–15)	8.39 ± 2.15 (6–13)
Type of repair • Retromuscular (sub-lay), n (%) • On-lay repair, n (%) • IPOM plus technique, n (%)	25 (54.3%) 21 (45.7%) -	- - 33 (100%)
Operative time, min (mean ± SD, range)	140 ± 22.4 (120–198)	144.4 ± 22.3 (119–196)
Peritoneal adhesion index (mean ± SD, range)	11.2 ± 6.8 (4–24)	10.8 ± 6.7 (3–23)

In the open surgery group, retromuscular (sub-lay) repair was successfully performed in 25 patients (54.3%). In the remaining 21 patients (45.7%), sub-lay repair was not feasible due to severe post-traumatic anatomical alterations, including disruption of the posterior rectus sheath, dense adhesions, and extensive scarring; therefore, an on-lay mesh repair was performed.

In the laparoscopic group, all patients underwent IPOM plus repair with successful primary fascial defect closure in all cases.

Extensive adhesiolysis was required in most patients in both groups, reflecting the high prevalence of severe intra-abdominal adhesions following damage control surgery. No statistically significant difference in operative time was observed between the open and laparoscopic groups (*p* > 0.05).

Within the open surgery group, no statistically significant differences were observed between patients undergoing sub-lay and on-lay repair with regard to operative time, length of hospital stay, postoperative pain scores, recovery of bowel function, or early postoperative complications.

### Length of hospital stay

The mean length of hospital stay in the open surgery group, taking institutional discharge requirements into account, was 16.89 ± 4.29 days (range, 10–25 days). In the laparoscopic group, the average duration of hospital stay was significantly shorter, amounting to 10.39 ± 4.34 days (range, 7–22 days). This difference between the groups was statistically significant (*p* < 0.001).

### Postoperative pain

Postoperative pain intensity was assessed using the visual analogue scale (VAS) on postoperative days (POD) 1, 2, and 3. In the open surgery group, mean VAS scores were 6.41 ± 0.83 on POD 1 and 5.59 ± 1.00 on POD 2. In the laparoscopic group, postoperative pain was significantly lower during the early postoperative period, with mean VAS scores of 5.71 ± 0.91 on POD 1 and 4.97 ± 1.09 on POD 2. The differences between the groups were statistically significant on both POD 1 (*p* < 0.001) and POD 2 (*p* < 0.05).

By POD 3, pain intensity decreased substantially in both groups and became comparable, with mean VAS scores of 3.93 ± 0.68 in the open group and 3.91 ± 0.68 in the laparoscopic group (*p* > 0.05).

### Recovery of bowel function

Recovery of bowel function was evaluated as the time to first postoperative bowel movement. In the open surgery group (n = 46), first postoperative bowel movement occurred on POD 2 in 16 patients (34.8%), on POD 3 in 22 patients (47.8%), and on POD 4 in 8 patients (17.4%). In the laparoscopic group (n = 33), recovery of bowel function occurred earlier. First bowel movement was observed on POD 2 in 20 patients (60.6%) and on POD 3 in 13 patients (39.4%). No patients in the laparoscopic group experienced bowel recovery later than on POD 3.

Overall, patients undergoing laparoscopic IPOM plus repair demonstrated significantly earlier recovery of bowel function compared with those treated with open surgery (*p* < 0.01).

Postoperative intestinal obstruction was observed in 1 patient (2.2%) in the open surgery group. The condition was managed conservatively, with resolution achieved without the need for surgical reintervention. No cases of postoperative intestinal obstruction were recorded in the laparoscopic group. The difference between the groups was not statistically significant (*p* > 0.05).

### Seroma formation

All patients underwent routine postoperative ultrasound examination of the anterior abdominal wall to detect seroma formation. Seromas were identified in 19 patients (41.3%) in the open surgery group and in 8 patients (24.2%) in the laparoscopic group. Although the incidence of seroma formation was lower following laparoscopic IPOM plus repair, the difference between the groups was not statistically significant (*p* > 0.05). In both groups, seromas were managed conservatively. Ultrasound-guided aspiration was performed when clinically indicated, and no patient required surgical intervention. In patients with clinically relevant seromas, hospital stay was prolonged until complete resolution was confirmed by follow-up ultrasound examination.

Comparison between patients with and without seroma formation revealed no significant differences in operative characteristics, including hernia defect size, extent of adhesiolysis, or number of previous laparotomies. In the open surgery group, seroma formation was not associated with mesh position (on-lay or sub-lay). In the laparoscopic group, all meshes were placed intraperitoneally according to the IPOM plus technique.

Therefore, the higher incidence of seroma formation observed after open repair is unlikely to be attributable to differences in hernia characteristics or mesh positioning. It is more likely related to the greater degree of soft tissue dissection and the creation of subcutaneous dead space inherent to open surgical techniques, particularly in patients with extensive post-traumatic scarring following damage control surgery.

### Surgical site infection

No cases of surgical site infection were observed in either group during the 90-day postoperative follow-up period. Specifically, no superficial, deep, or organ space infections were diagnosed according to clinical assessment and institutional diagnostic criteria.

### Non-surgical complications

No non-surgical postoperative complications were observed in either group during the 90-day follow-up period. Specifically, no cases of cardiovascular events, respiratory complications, thromboembolic events, urinary tract infections, or other systemic complications were recorded.

### Readmission and reoperation

No patients in either group required reoperation within the 90-day postoperative period. Similarly, no hospital readmissions were recorded in either the open surgery or laparoscopic IPOM plus group during follow-up.

A comparative summary of all postoperative outcomes is provided in Table 3.

**TABLE 3:** Postoperative outcomes comparison.

Parameter	Open group (n = 46)	Laparoscopic group (n = 33)	p-value
Length of hospital stay, days (mean ± SD, range)	16.89 ± 4.29 (10–25)	10.39 ± 4.34 (7–22)	<0.001
First postoperative bowel movement, n (%) • POD 2 • POD 3 • POD 4	16 (34.8%) 22 (47.8%) 8 (17.4%)	20 (60.6%) 13 (39.4%) 0	<0.01
VAS pain score, mean ± SD • POD 1 • POD 2 • POD 3	6.41 ± 0.83 5.59 ± 1.00 3.93 ± 0.68	5.71 ± 0.91 4.97 ± 1.09 3.91 ± 0.68	<0.001 <0.05 >0.05
Postoperative complications, n (%) • Seroma formation • Postoperative small bowel obstruction	20 (43.5%) 19 (41.3%) 1 (2.2%)	8 (24.2%) 8 (24.2%) 0	>0.05 >0.05 >0.05

## Discussion

The present study evaluated early outcomes of elective ventral hernia repair in patients who had previously undergone surgery for abdominal shrapnel injuries managed according to damage control surgery (DCS) principles. Damage control strategies have markedly improved survival in patients with severe abdominal trauma [1, 2]. However, this approach frequently results in substantial disruption of the abdominal wall, leading to the development of large and complex ventral hernias. In our cohort, all patients had W3 defects according to the classification of the European Hernia Society, underscoring the significant reconstructive challenge in this population [3].

Abdominal wall reconstruction after DCS differs fundamentally from elective ventral hernia repair in non-trauma settings. Multiple laparotomies, temporary abdominal closure, secondary wound healing, and dense adhesions commonly distort anatomical planes and compromise the posterior rectus sheath. Although retromuscular (sub-lay) repair is generally regarded as the preferred technique for large ventral hernias [4, 5], our findings confirm that this approach is not consistently feasible following DCS. In nearly half of the patients in the open group, the retromuscular plane was partially or completely disrupted, necessitating on-lay mesh placement. Importantly, no significant differences in short-term outcomes were observed between sub-lay and on-lay repairs within the open cohort, suggesting that in the presence of trauma-related anatomical distortion, intraoperative feasibility may outweigh the theoretical advantages of mesh position.

The introduction of laparoscopic IPOM plus repair provided an alternative reconstructive strategy in this complex setting. Evidence from non-trauma populations indicates that IPOM plus is associated with reduced postoperative pain and shorter hospital stay compared with open repair [6, 7]. However, data specifically addressing patients after DCS remain limited. In the present study, laparoscopic IPOM plus repair was feasible despite extensive adhesions and post-traumatic anatomical changes, provided that careful adhesiolysis and secure fascial closure were achieved.

Although laparoscopic IPOM plus was introduced later during the study period, the operating surgeons had prior experience with this technique in elective ventral hernia repair outside the present study cohort. Review of the first and last 10 laparoscopic cases did not reveal any obvious differences in operative time, length of hospital stay, postoperative recovery, or complication rates. Nevertheless, given the complexity of post-traumatic abdominal wall reconstruction after DCS, a learning-curve effect specific to this patient population cannot be completely excluded.

A significant reduction in length of hospital stay was observed in the laparoscopic group. Although discharge criteria in our institution require complete wound healing and suture removal, patients undergoing IPOM plus were discharged approximately 6 days earlier than those treated with open surgery. This finding is consistent with systematic reviews demonstrating shorter hospitalisation following minimally invasive ventral hernia repair [7, 8]. Nevertheless, the duration of hospital stay may also have been influenced by local institutional factors and discharge policies. Reduced access trauma, avoidance of extensive subcutaneous dissection, and smaller wound surfaces are likely to have contributed to faster overall recovery.

Postoperative pain was significantly lower in the laparoscopic group during the early postoperative period (POD 1–2), although the difference was no longer evident by POD3. These findings are in keeping with published meta-analyses reporting lower early pain scores after laparoscopic IPOM plus compared with open techniques [6, 7]. In patients with a history of multiple laparotomies, limiting additional surgical trauma may be particularly important in facilitating early mobilisation and functional recovery.

Recovery of bowel function occurred earlier following laparoscopic repair. As comparative data in post-DCS trauma populations are scarce, this observation should be interpreted cautiously. Nevertheless, the reduced abdominal wall dissection and potentially lower inflammatory response associated with minimally invasive surgery may contribute to earlier restoration of gastrointestinal function. Further prospective studies are required to clarify this relationship in trauma cohorts.

Seroma formation was more frequent after open repair, although the difference did not reach statistical significance. The observed incidence falls within the expected range for large ventral hernia reconstruction [4, 7]. In our cohort, seroma development was not associated with hernia size, extent of adhesiolysis or mesh position. The higher rate following open repair is likely related to wider soft tissue dissection and the creation of subcutaneous dead space, which are inherent to open techniques, particularly in patients with extensive post-traumatic scarring.

No surgical site infections, systemic complications, reoperations or readmissions were recorded within the 90-day follow-up period in either group, supporting the safety of elective reconstruction in this setting.

Within the limitations of this retrospective single-centre study, the introduction of laparoscopic IPOM plus repair in patients undergoing abdominal wall reconstruction after DCS was associated with shorter hospital stay, reduced early postoperative pain and earlier recovery of bowel function, without an increase in short-term complications. These findings suggest that laparoscopic IPOM plus represents a valuable and effective alternative to open repair in this challenging post-traumatic population.

### Strengths

This study has several important strengths. It addresses a clearly defined and underrepresented patient group - individuals with large post-traumatic ventral hernias following damage control surgery for combat-related abdominal shrapnel injuries.

All procedures were performed in a single specialised military centre using standardised perioperative protocols, ensuring consistency in surgical technique and perioperative care. The cohort was clinically homogeneous, as all patients had comparable injury mechanisms, surgical histories and defect characteristics, and the exclusion of significant comorbidities minimised potential confounding.

The comparison reflects a structured transition from open repair to laparoscopic IPOM plus within the same institution and during consecutive time periods, thereby limiting selection bias and maintaining baseline comparability between groups.

Early postoperative outcomes were assessed comprehensively, including pain scores, recovery of bowel function, seroma formation confirmed by routine ultrasound, and 90-day reoperation and readmission rates, with no missing data. These features strengthen the reliability of the findings and support the validity of the short-term conclusions.

### Limitations

The main limitation of this study is the relatively short and unequal follow-up between groups, which precluded the assessment of long-term outcomes such as hernia recurrence and chronic pain. As a result, the present analysis is restricted to early postoperative results and does not allow conclusions regarding the durability of repair.

Second, the retrospective design carries an inherent risk of selection bias and limits control over potential confounding variables. Although baseline characteristics were comparable between groups, unrecognised factors may have influenced treatment allocation and outcomes. An additional limitation relates to the chronological nature of group allocation. Open repairs were performed during an earlier study period, whereas laparoscopic IPOM plus became the standard approach later. Consequently, the comparison reflects a sequential change in clinical practice rather than concurrent treatment allocation. Although perioperative management protocols remained unchanged throughout the study period, unmeasured temporal factors such as increasing institutional experience, improvements in perioperative care pathways, or changes in organisational efficiency may have influenced some outcomes, particularly length of hospital stay. Therefore, the observed differences cannot be attributed exclusively to the surgical technique.

In addition, laparoscopic IPOM plus was introduced later during the study period. Although the surgeons had prior experience with this technique in non-trauma patients, its application in patients with extensive post-traumatic adhesions and distorted abdominal wall anatomy represents a distinct technical challenge. Therefore, a learning-curve effect specific to this patient population cannot be completely excluded.

Third, this was a single-centre study conducted in a specialised military institution and included exclusively male patients with combat-related injuries. Consequently, the findings may not be fully generalisable to civilian populations or to patients with ventral hernias of non-traumatic origin.

Another limitation is that the open surgery cohort included both sub-lay and on-lay repairs. However, no significant differences in early postoperative outcomes were identified between these subgroups within the present study.

Finally, the sample size was relatively modest, which may have limited the statistical power to detect differences in less frequent complications.

Prospective multicentre studies with longer and standardised follow-up are required to confirm these findings and to determine whether the early advantages observed after laparoscopic IPOM plus repair translate into sustained long-term benefit.

### External validity and future directions

Despite its limitations, this study suggests that laparoscopic IPOM plus repair may offer meaningful early benefits for patients with large ventral hernias following damage control surgery for abdominal shrapnel injuries. The observed reductions in hospital stay, early postoperative pain and time to restoration of bowel function are clinically relevant in a population characterised by extensive adhesions and distorted anatomy. The technique proved technically feasible in this demanding setting and may be considered in other centres managing similar complex post-traumatic reconstructions.

The findings must, however, be interpreted within the specific context in which the study was conducted. The cohort was treated at a single military hospital in Ukraine during wartime and consisted exclusively of male patients without major comorbidities. Institutional discharge policies also resulted in longer hospital stays than would typically be expected in other healthcare systems. In addition, the injury pattern, characterised by combat-related shrapnel trauma, multiple prior laparotomies and extensive scarring, differs substantially from that encountered in most civilian ventral hernia practice. For these reasons, caution is required when applying the present results to non-military populations, women, older patients, individuals with significant comorbidity, or routine incisional hernia repair. The findings should therefore be regarded as context-specific rather than broadly generalisable.

Nevertheless, several observations are likely to have wider relevance. These include the frequent difficulty of achieving retromuscular repair after damage control surgery, the comparable short-term outcomes of on-lay and sub-lay techniques in open reconstruction, and the feasibility of laparoscopic IPOM plus repair with fascial closure even in the presence of severe adhesions. Such considerations may be applicable to surgeons managing post-open abdomen or trauma-related hernias in other settings.

Future research should seek to confirm these findings in prospective, multicentre studies with larger sample sizes and standardised long-term follow-up. Assessment of hernia recurrence, chronic pain and quality of life will be essential to determine whether the early advantages observed translate into sustained clinical benefit. Inclusion of more diverse patient populations, including civilian trauma cases and individuals with relevant comorbidities, would further clarify the external validity of these results.

In this retrospective single-centre study of patients with large ventral hernias following damage control surgery for abdominal shrapnel injuries, laparoscopic IPOM plus repair was shown to be feasible and safe in the early postoperative period. Compared with open repair, the laparoscopic approach was associated with a shorter hospital stay, reduced early postoperative pain and more rapid recovery of bowel function, without an increase in short-term complications.

Open retromuscular repair was not consistently achievable in this post-traumatic setting because of disruption of anatomical planes and dense adhesions. When retromuscular reconstruction was not feasible, on-lay repair provided comparable short-term results within the open cohort. These findings underline the importance of tailoring the reconstructive strategy to intraoperative anatomical conditions rather than adhering rigidly to a single preferred technique.

Although the results are limited to early outcomes and reflect the experience of a specialised military centre, they suggest that laparoscopic IPOM plus may represent a valuable alternative for abdominal wall reconstruction after damage control surgery. Further prospective studies with long-term follow-up are required to determine whether the early advantages observed translate into durable clinical benefit.

## Data Availability

The raw data supporting the conclusions of this article will be made available by the authors, without undue reservation.
